# A Model Based Security Testing Method for Protocol Implementation

**DOI:** 10.1155/2014/632154

**Published:** 2014-07-06

**Authors:** Yu Long Fu, Xiao Long Xin

**Affiliations:** ^1^LIUPPA, University of PAU and Academy of Bordeaux, 40000 Mont de Marsan, France; ^2^Department of Mathematics, Northwest University, Xi'an 710069, China

## Abstract

The security of protocol implementation is important and hard to be verified. Since the penetration testing is usually based on the experience of the security tester and the specific protocol specifications, a formal and automatic verification method is always required. In this paper, we propose an extended model of IOLTS to describe the legal roles and intruders of security protocol implementations, and then combine them together to generate the suitable test cases to verify the security of protocol implementation.

## 1. Introduction

Since the former employee of CIA, Edward J. Snowden, reveals the global surveillance of National Security Agency (NSA) [1], the suspicions over the existing security mechanisms and products spread over the world. As a consequence, the cryptographic scientists may need to reverify the popular cryptography methods and protocol designs (RSA, DES, etc.). The documents revealed from Snowden also imply the back-doors may exist in some popular network devices/services, such as the productions from CISSCO, IBM, Microsoft, and Google, which recalls the importance of the security verification over the network productions (including hardware and software if the interactions between the possible intruders and the protocol implementations will not result in any leakage of privacy, the protocol implements are considered as secure). This is because, even when the security protocols are designed perfectly, the back-doors may be also proposed during the implementing of the productions and may make the privacies leak to some potential attackers. In this paper, we are concerned with this important security problem and propose the model and algorithms to verify the security of protocol implementations automatically.

Security protocols are communication protocols, which guarantee the securities (authentication or confidentiality) by the defined rules and cryptography methods [2]. A protocol implementation is a solid solution of the corresponding protocol specification, which can be one or a part of software/hardware, and is supposed to work as the designs of the protocol. The implementations of security protocol are usually separated into different network devices and are in charge of the security functions to guarantee the communication security. As the importance to those implementations, those declared security functions should be well verified. However, the classic verification method of protocol implementation, the* protocol testing* [3] methods, is not suitable for security verifications. Although several methods have been proposed in the literature [4–6], the problem of security verification over protocol implementations is still an open problem.

*Related Works*. Recently, the problems of model based security verifications on protocol implementations have attracted more and more attention of academy. Based on the developing security reasoning theorems (PCL [7], Pi-Calculus [8], and HLPSL [9]) and the corresponding automatic model checking approaches (the methods described in [10–12]), the authors of [4] came up with an approach to verify the security of protocol implementation. They use model checking methods to automatically generate the test cases, which contain the possible security flaws of the protocol, and then execute those test cases on the implementations to verify their security. Although the presented approach fills the gaps between “security protocol analysis” and “protocol implementation verification” to some degree, several problems are still noticed. First of all, the presented model checker is based on ASLan, which is a specific protocol description language, and makes the translation to other model checkers become difficult. Secondly, the generated test cases only contain the counterexamples related to the detected security flaws; other possible messages sequences which can also cause the insecurity (e.g., the message sequences contain some wrong messages formats) are not included. In [5], the authors proposed a mutation based test generation method for security protocol implementation verification. They propose several mutation rules over the conformed messages sequences of the protocol specification to construct the possible counterexamples. Although the concepts of mutations and fuzzy sets [13] are quite useful to simulate the artificial intelligence of computer network, it is difficult to decide the completeness of the proposed mutator.

*Paper Contribution*. In this paper, by investigating the features of security protocols and the limitations of the existing protocol testing methods, we propose a security extension of Input/Output Transition System to model and test the protocol implementation. The proposed model inherits the clarity of finite automata and can describe the security properties and most of the protocol behaviors with the definition of transition. We also consider the possible network intruder as one part of the system and propose a model to simulate the attack actions. Then, by the proposed models, the complex security protocol actions can be presented in the way of sequence transitions of the reachable graphic model. The possible test cases can be generated through the model with the proposed algorithm.

The following sections are organized as follows. In Section 2, we briefly introduce the used protocol testing method IOLTS and some testing theories over it. In Section 3, from the requirements of security protocol, we analyze the limitations of the existed IOLTS model and present an extended model of IOLTS under some required assumptions. Then, in Section 4, those modeled protocol components are combined as one system and an intruder model is proposed to simulate the malicious attack actions. The test approach and test case generation algorithms are also presented in this section. And finally, in Section 5, we conclude this paper and introduce some future works.

## 2. Protocol Testing Theory

Protocol testing represents a set of automata based modeling and testing methods to verify the correctness of the protocol implementations, which has been proposed and widely accepted in the industries since Gonenc [14] found the distinguishing sequences in 1970 [3]. Through the serious preset inputs, the unobservable system status and some internal actions can be deduced by checking the corresponding outputs; then the protocol implementations can be verified by the test execution. This verification method has been used to solve many problems, such as conformance [15], robustness [16], web service security [17], and cloud computing security [18].

There are several models existing in protocol testing methods. But in this paper, we only consider the model of IOLTS. An IOLTS takes the system behavior as labels and serves as a semantic model for various formal specification languages [19]. Normally a finite IOLTS system can be described as a 4-tuple array.

**Definition 1 (labeled transition system).**

A labeled transition system is a 4-tuple array 〈*S*, *L*, *T*, *s*_0_〉 where

- *S* is a finite set of states;
- *L* is a finite set of labels, which contains two subsets: input labels *L*_*I*_ and output labels *L*_*U*_;
- *T* is the transition relation, where *T*⊆*S* × (*L* ∪ {*τ*}) × *S*;
- *s*_0_ is the initial state.

If ∃*a* ∈ *L*_*I*_, then we write ?*a* to represent that *a* is an input label; if ∃*b* ∈ *L*_*U*_, then we write !*b* to identify that *b* is an output label; we have *L*_*I*_∩*L*_*U*_ = *∅*, *L*_*I*_ ∪ *L*_*U*_ = *L*. If the input and output labels are not identified, the model is labeled transition system (LTS). *τ* denotes the internal and unobservable (to the tester) actions of the system. A trace means a finite sequence of the observable actions; they are going to be used as test sequences to verify the system. If *σ* ∈ L* (L* = *L* ∪ {*τ*}), then |*σ*| denotes the length of trace of *σ*. If *q* ∈ *S*, then *Out*(*q*) denotes all the output labels from *q*, *In*(*q*) denotes all the input labels to *q*, and *Out*(*S*, *σ*) denotes the output of *S* after *σ*. We denote the class of all IOLTS over *L*_*I*_ and *L*_*U*_ by *IOTS*(*L*_*I*_, *L*_*U*_) or *LTS*(*L*_*I*_ ∪ *L*_*U*_).

**Definition 2 .**

Let *P* = 〈*S*, *L*, *T*, *s*_0_〉 be a labeled transition system, *s* and *s*′ ∈ *S*, and let *μ*_*i*_ ∈ *L* ∪ {*τ*}, *a*_*i*_ ∈ *L*, and *σ* ∈ L*; then we have the following notations:

(1) s⟶μs′=def ∃t∈T, t=(s,μ,s′), t.pre=s, t.nex=s′, t.lab=μs→μ1⋯μns′=def ∃s0,…,sn:s=s0⟶μ1s1⟶μ2⋯⟶μnsn=s′s→μ1⋯μn=def ∃s′:s→μ1⋯μns′s⇒ϵs′=def s=s′ or  s⟶τ⋯τs′s⇒as′=def ∃s1,s2:s⇒ϵs1⟶as2⇒ϵs′s⇒a1⋯ans′=def ∃s0⋯sn:s=s0⇒a1s1⇒a2⋯⇒ansn=s′s⇒σ=def ∃s′:s⇒σs′trace(p)=def {σ∈L∗ ∣ p⇒σ}init(p)=def {a∈L ∣ p⇒a}.

A test case *te* is a specification for the behaviours of a tester, which exists in an experiment to be carried out with an implementation under test (IUT). Such behaviour, like other behaviours, can be specified by an IOLTS [20]. Then the test case *t* can be described as an IOLTS over some specific action set *L*_*I*_ and *L*_*U*_ and with a test verdict (*pass* or* fail*) in the end (*te* : *i* → {*fail*, *pass*}, *i* ∈ *I* *O* *LTS*(*L*_*I*_, *L*_*U*_)). In other words, a test case is a set of transition sequence end with test verdict. Generally, the considered actions of the test cases must relate to the specific protocol specification.

## 3. Transition Models on Security Protocols

Although the IOLTS based testing methods have been proved to be useful in verifying the protocol implementations, it cannot be used to verify the correctness of security protocol implementations, because of some important features of security protocol. Comparing with the normal system protocols, security protocols have the following important features which stop the modeling by IOLTS.

- Security properties: security protocols usually need to consider security properties such as nonce and session id to help the security functions to identify the security. Those security properties are usually the contents of the exchanging or received messages and are not considerable in IOLTS system.
- Security checking functions: security protocols use several predefined security rules (e.g., comparing the received nonce with the holding one) to verify the security, which are called security checking functions here. Those fundamental security functions are hard to model by IOLTS. Someone may say that the internal action *τ* of IOLTS can present those functions, but considering that the security properties cannot be distinguished, an input of IOLTS will trigger multiply checking functions and it is meaningless to the testing.
- Multiple roles: security protocols naturally contain multiple roles (at least one initiator and one responder), while the IOLTS is designed for one system component.
- Intruder: most of the security protocols are designed to avoid the attacks from intruder, which is considered as participation of protocol. Notice that this intruder is not presented in the security protocol specification, and it is impossible to propose a model with IOLTS.

In order to describe the required features, we propose extensions of IOLTS with several considerations. To identify the security properties, we define the security properties as “partial label” of the inputs actions and define “parsing state” and “combination state” to construct the transition. This transition then is going to trigger the security checking functions. Secondly, we impact the way of IO automata [21], which defines the internal actions *τ* with different suffix to identify different internal actions to this extended model. The security checking functions which are triggered by the proposed “partial label transition” can be identified. The methods of solving the problems of “multiple roles” and “intruder” are going to be presented in the next section. Then, we give a model of security extended labeled transition system (SE-LTS) model as follows.

**Definition 3 .**

(SE-LTS) A security extended labeled transition system is an extension of IOLTS for the components of security protocol; it is a 4-tuple array 〈*s*_0_, *S*_*se*_, *L*_*se*_, *T*_*se*_〉, where

- *s*_0_ is the initial state;
- *S*_*se*_ is a set of states and *S*_*se*_ = *S*_*n*_ ∪ *S*_*p*_, where *S*_*p*_ is a set of “parsing states,” which parses the received input action into partial labels and *S*_*n*_ represents the set of other normal states;
- *L*_*se*_ is a finite set of labels and {*l* ∈ *L*_*se*_∣*l* = *l*_0_ · *l*_1_ ⋯ *l*_*n*_}, where *l*_*n*_ is one partial label of *l* and · represents the function of concatenation. We define *L*_*p*_ as a finite set of partial labels of all labels in *L*_*se*_; then ∀*l*_*n*_ ∈ *L*_*p*_. The unobservable internal actions are marked as *τ*_*n*_, which indicates different internal actions by the suffix. The set of internal actions is marked as Θ;
- *T*_*se*_ is the transition relation, *T*_*se*_⊆{*S*_*n*_ × *L*_*se*_ × *S*_*p*_}∪{*S*_*p*_ × *L*_*p*_ × *S*_*n*_}∪{*S*_*n*_ × Θ × *S*_*c*_}∪{*S*_*c*_ × *L*_*se*_ × *S*_*n*_}.

A partial label is one part of system action which is sufficient to trigger an internal action of the system. A system action may contain one or multiple partial labels.

**Definition 4 .**

Let *P* = 〈*s*_0_, *S*_*se*_, *L*_*se*_, *T*_*se*_〉 be a SE-LTS with *s*, *s*′ ∈ *S*_*se*_, and let *μ*_*i*_ ∈ *L*_*se*_ ∪ Θ, *a*_*i*_ ∈ *L*_*se*_, and *a*_*i*_ = {*a*_*i*__0_ · *a*_*i*__1_ ⋯ *a*_*i*__*n*_}; we have the following notations:

(2) s⟶μs′=def ∃t∈T,t=(s,μ,s′), t.pre=s, t.nex=s′, t.lab=μs→μ1⋯μns′=def ∃s0,…,sn:s=s0⟶μ1s1⟶μ2⋯⟶μnsn=s′s→μ1⋯μn=def ∃s′:s→μ1⋯μns′s⇒as′=def ∃s1,s3,…,s2n+1∈Sp∪Sc,s2,s4,…,s2n+2∈Sn:s⟶as1⟶a0s2⟶τ0⋯s2n+1⟶ans2n+2⟶τns′Parse(a)=def {a0,a1,…,an}.

**Example 5 .**

The Needham-Schroeder Public Key (NSPK) protocol [22] is an asymmetric cryptography based authentication protocol, which defines the handshakes between two participants: the initiator *i* and the responder *r*. The brief protocol narration can be presented with the three message exchanges as follows:

(3) $$\begin{array}{c} \text{Msg}\;1.\;i⟶r\;\left(\text{Ask}\right):{\left\{n_{i},i\right\}}_{pk_{r}}\\ \text{Msg}\;2.\;r⟶i\;\left(\text{Rpl}\right):{\left\{n_{i},n_{r}\right\}}_{pk_{i}}\\ \text{Msg}\;3.\;i⟶r\;\left(\text{Cfm}\right):{\left\{n_{r}\right\}}_{pk_{r}}. \end{array}$$

The NSPK protocol assumes the nonces *n*_*i*_ and *n*_*r*_ are completely random and only the one who holds the private key can decrypt the cipher encrypted by the public key. In this case, when the unique *n*_*i*_ is sent to *r* through* Msg1*, although this message may be captured by some intruders, the *n*_*i*_ can be only obtained by *r*. Similar to the nonce *n*_*r*_ through message* Msg2*, it can be only obtained by *i*. Then these unique nonces are used to verify the correctness and authentication of the protocol participations.

**Figure 1:**
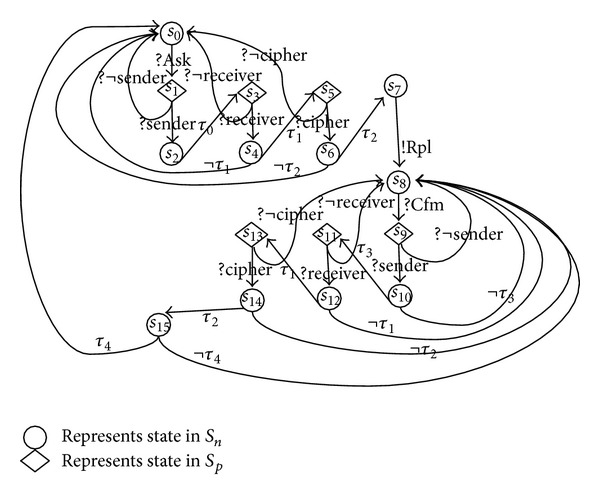
SE-LTS for NSPK receiver.

It is hard to model and analyze the security issues of security protocols by using the general models of automata, because the important security properties are usually hidden inside the exchanging messages. However, with our SE-LTS model, the authentication processes can be modeled as internal actions and the important security properties are identified and modeled as partial labels, which are going to be used to trigger those internal actions of the protocol participations.  Figure 1 presents the receiver of NSPK protocol described by SE-LTS. In this example, *S*_*n*_ = {*s*_0_, *s*_2_, *s*_4_, *s*_6_, *s*_8_, *s*_10_, *s*_12_, *s*_14_, *s*_15_}, *S*_*p*_ = {*s*_1_, *s*_3_, *s*_5_, *s*_7_, *s*_9_, *s*_11_, *s*_13_}, and *S*_*c*_ = {*s*_7_}; *L*_*se*_ = {?*Ack*, !*Rpl*, ?*Cfm*}, where ?*Ack* = {?*se* *n* *d* *e* *r* · ?*receiver* · ?*cipher*}, ?*Cfm* = {?*se* *n* *d* *e* *r* · ?*receiver* · ?*cipher*}; Θ = {*τ*_0_, *τ*_1_, *τ*_2_, *τ*_3_, *τ*_4_}, where *τ*_0_ means *r* records the sender id; *τ*_1_ means *r* verifies the receiver id in ?*Ack* message equal to its id; *τ*_2_ means a successful description; *τ*_3_ means the received sender id in ?*Cfm* is equal to the recorded in ?*Ack*; *τ*_4_ means the received decrypted message is equal to *n*_*r*_. The mark of “¬” means the conditions inverse.

## 4. Networked Model and Intruder

### 4.1. Networked Model for Multiple Components

The security protocol implements usually are installed in distributed network devices. To present the connected feature of the security protocol, we use SE-LTS model to describe each form of participation first and then combine them together as a networked transition system. As the method proposed in [23], a communication medium is needed to glue those components. The normal state *s* ∈ *S*_*n*_ of *LTS*_*se*_(*L*) is defined with two levels:

- higher_level state *s*_*i*__*u* connects to the environment or other states of the same component;
- lower_level state *s*_*i*__*l* connects to the states of other components.

Then a common medium is considered by such transition, which begins from the lower_level state of one component and ends with the lower_level of initial state of another component. *S*_*i*_, *L*_*i*_ denote the states and labels in *LTS*_*se*_(*L*_*i*_), and *S*_*j*_ and *L*_*j*_ denote the state and labels in *LTS*_*se*_(*L*_*j*_); then if ∃!*l* ∈ *L*_*i*_, ∃*s*_*i*_ ∈ *S*_*i*_, !*l* ∈ *Out*(*s*_*i*_), and ∃*s*_*j*_ ∈ *S*_*j*_, ?*l* ∈ *L*_*j*_, ?*l* ∈ *In*(*s*_*j*_), the transition of the common medium between *I* *O* *LTS*_*i*_ and *I* *O* *LTS*_*j*_ is presented as $s_{i}\_l\overset{!l}{\to }s_{0}\_l$. We make *S*_medium_ to denote all the states and *T*_medium_ to denote all the transitions in the medium.

**Definition 6 (networked SE-LTS).**

The implementation of a security protocol contains a set of SE-LTS systems (*LTS*_*SE*_(*L*_*n*_); *L*_*n*_ represents the set of labels of the *n*th components, *n* = 0,1⋯), where those systems are connected sequently by some transitions of the medium. It is also a four-tuple array: 〈*s*_0__*sp*_, *S*_*sp*_, *L*_*sp*_, *T*_*sp*_〉, where

- *s*_0__*sp*_ is the set of initial states of *LTS*_*SE*_(*L*_*n*_), *n* = 0,1⋯;
- *S*_*sp*_ is a set of states and *S*_*sp*_ = *S*_*LTS*_*SE*_(*L*_0_)_ ∪ *S*_*LTS*_*SE*_(*L*_1_)_ ∪ ⋯∪*S*_*LTS*_*SE*_(*L*_*n*_)_ ∪ *S*_medium_;
- *L*_*sp*_ is a finite set of labels, *L*_*sp*_ = *L*_*LTS*_*SE*_(*L*_0_)_ ∪ *L*_*LTS*_*SE*_(*L*_1_)_ ∪ ⋯∪*L*_*LTS*_*SE*_(*L*_*n*_)_;
- *T*_*sp*_ is the transition relation, *T*_*sp*_ = *T*_*LTS*_*SE*_(*L*_0_)_ ∪ *T*_*LTS*_*SE*_(*L*_1_)_ ∪ ⋯∪*T*_*LTS*_*SE*_(*L*_*n*_)_ ∪ *T*_medium_.

**Example 7 .**

**Figure 2:**
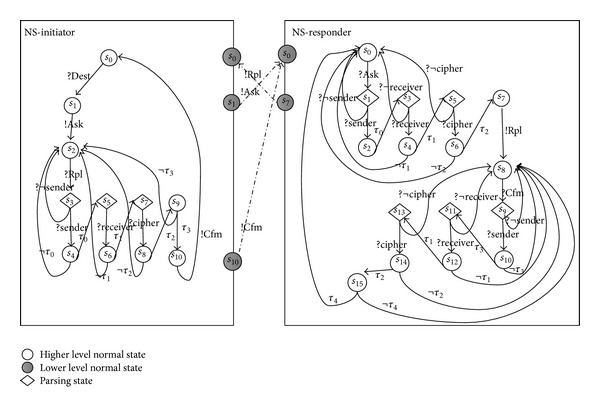
Glued security extension graph of NSPK.

The networked model of NSPK protocol can be presented as in Figure 2. The transitions between two communications are the medium transition. The lower_interface and higher_interface represent the same state.

### 4.2. Intruder of Security Protocol Communication

With the help of networked SE-LTS model (noted as *N* *LTS*_*se*_(*L*)), the specification of security protocol can be modeled as transition system. The sequential actions designed in security protocols are modeled as transition sequences of the presented reachable graph. Now, we need to consider the following question: “what kind of test cases (transition sequences) can be used to verify the security of the protocol implementations?” To address this, we give a soundness definition of the security of protocol implementations.

**Definition 8 (soundness security of protocol implementation).**

If the interactions between the possible intruders and the protocol implementations will not result any leakage of privacy, the protocol implements are considered as secure.

The privacies are the values of some security properties, such as nonce and private key. Then the problem changes to be “how to calculate the possible transition traces which contain the interactions between intruder and protocol implementations” and “how to distinguish the leakage of privacy.” To address these questions, we need to simulate the intruder model in the proposed networked SE-LTS model.

#### 4.2.1. Testable Intruder Model

An intruder is a powerful agent, which may participate in the protocol executions but with a purpose to attack the system. An intruder usually pretends as a legal role and has the abilities to eavesdrop, insert, or intercept the communicating messages between the legal agents. An intruder usually executes a man-in-the-middle attack. We consider the network intruder with the following assumptions.

1. Dolev and Yao intruder assumption: the Dolev and Yao intruder assumption was proposed since 1983 with two main properties [24]:
  - the cryptography is assumed to be perfect: a message can only be decrypted by someone who has the proper key (there is no way to crack the scheme);
  - the messages are considered to be abstract terms: either the intruder learns all messages inside the encryption (because he has the key) or he learns the encrypted message.
2. Tester is powerful: a tester of the system has more permissions; a tester can operate the exchanging message as the proposed intruder assumption.

And a network intruder has the following features:

- no refusal: an intruder can pretend as any legal roles of the security protocol and participate in the protocol execution. It accepts all types of output messages from any legal/illegal participations and gives the corresponding response messages (including attack messages);
- knowledge learning: an intruder can parse the received messages, analyze them, and update its knowledge. This knowledge may contribute to the generation of an executable attack message;
- message matching: the output messages of the intruder are not generated randomly; they are going to be accepted by the destined principle. The outputs are generated through the intruder knowledge and will match the format of the input messages of the destination.

The intruder model needs to present those listed features and must be compatible with the models of the specifications. According to our assumption, the testable intruder is under control of the tester, so all of its functions (including the security functions) are reachable and observable to the tester. Here we simply use two categories of actions:* forward* and* modify*, which are triggered by the partial labels of the received messages. An intruder model of SE-LTS is defined as follows.

**Definition 9 (testable intruder model).**

A testable intruder model of a specific protocol 〈*s*_0__*sp*_, *S*_*sp*_, *L*_*sp*_, *T*_*sp*_〉 is a SE-LTS system, which is a 4-tuple array 〈*s*_0_, *S*_*int*_, *L*_*int*_, *T*_*int*_〉, where *L*_*int*_∩*L*_*sp*_ = *L*_*U*__*sp*_ and ∀*a*_*i*_ ∈ *L*_*I*__*int*_, ∀*a*′ ∈ *P* *a* *r* *se*(*a*_*i*_), ∃*s*, *s*′ ∈ *S*_*int*_,

(4) $$\begin{array}{c} s\overset{a^{\prime }}{⟶}\overset{\left\{foward,modify\right\}}{\to }s^{\prime }. \end{array}$$

**Figure 3:**
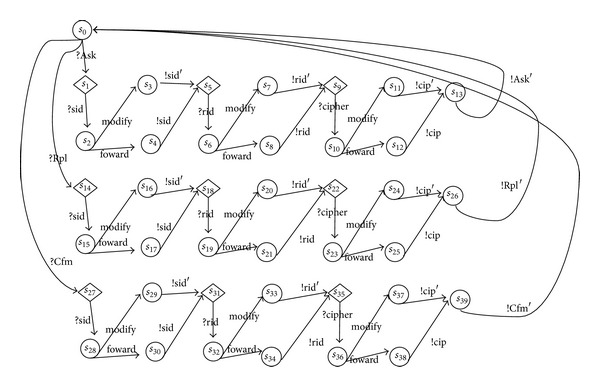
Intruder model of NSPK.

An example of testable intruder model of NSPK protocol is presented in Figure 3.

#### 4.2.2. Combination of the Intruder Model and the Networked SE-LTS

**Figure 4:**
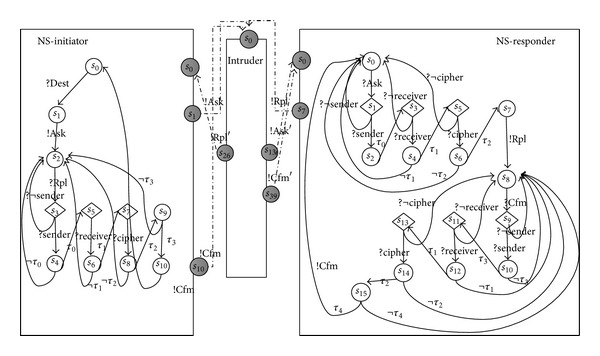
Intruder contained model, NSPK.

In order to calculate the possible transitions related to the intruder actions, we consider the intruder models that exist inside the communication medium. In this case, all the related actions to the security of protocols specifications are modeled as transitions of one combined transition system and can be presented in one reachable graph. For the example of NSPK protocol, the finial result of reachable graph is presented in Figure 4. The used intruder model is presented in Figure 3. We denote this combination model as *C* *N* *LTS*_*se*_(*L*_*sp*_). Generally, in a security protocol, once the current security session finishes, the security implementations will always prepare for the next session. So the transition traces of each session of the protocol implementations usually begin and end with initial states. Meanwhile, if the received message is wrong, the implementation will go back to the waiting state and wait for the correct message. Those two kinds of situations should be both considered by the test cases, and we say* a security test case is a self-complete transition trace, which begins from the initial state and ends with the state which is waiting for the inputs from other components*. We say a transition sequence is self-complete if its input actions, which are satisfied by the output of other components, are also included in this transition trace.

#### 4.2.3. Test Cases Generation and Verification

As the model *C* *N* *LTS*_*se*_(*L*_*sp*_) can be presented with a reachable graph, the problem of calculating the required traces can be solved by searching traces in graph and it is NP-complete. Here we propose a “deep first search algorithm” to calculate the possible traces automatically. Similar to the method we used in [25], the proposed algorithm calculates the traces from the possible end state to the initial state. The pseudocodes are presented in Algorithm 1.

**Proof**

The reason of the correctness of Algorithm 1 can be shown inductively as follows.

- A basic transition machine M only contains one transition *t*. Then by following the algorithm of “Traceback” *trace*_*i*_ = {*t*}; because *t*.*pre* is initial state, the Checkglue is called. Because M only contains one transition, *t*.*stimuli* is empty. And “check_glue” ends; then “Traceback” ends. The transition trace is completely found and recorded.
- In general, we can assume that the first *n* − 1 transitions of* M* are traced after *n* − 1 iterations of “Traceback.” Then *trace*_*i*_ = {*t*_0_, *t*_1_ ⋯ *t*_*n*−1_}. ∀*t* ∈ *trace*_*i*_, *t*.*stimuli* is empty.
- The transition *t* = *t*_*n*_ is taken as inputs of “Traceback” algorithm. By following the algorithm, *t* is recorded in *trace*_*i*_, then *trace*_*i*_ = {*t*_0_, *t*_1_ ⋯ *t*_*n*−1_, *t*_*n*_}; *t*.*pre*_*s* *t* *a* *te* is initial state; then “check_glue” is recalled. According to the last step, the first *n* − 1 elements of *trace*_*i*_ do not have stimuli transition. We only need to check whether *t*_*n*_ has stimuli transition. Because *t*_*n*_ is the last transition, if *t*_*n*_.*stimuli* is not empty, then another Traceback will be called, and *t*_*n*_ will not be the last transition. So *t*_*n*_ must not have stimuli transition; then *trace*_*hassti* is empty. The “check_glue” ends, and “Traceback” ends. *trace*_*i*_ = {*t*_0_, *t*_1_ ⋯ *t*_*n*−1_, *t*_*n*_}. All the transitions of* M* traveled.

By using this proposed algorithm through *C* *N* *LTS*_*se*_(*L*_*sp*_), the transition traces related to the security of protocol implementations (denoted as *Tr*_*gen*_) can be calculated. Then we need to decide the* test verdict* for each transition trace to obtain the security test cases. According to the soundness security of Definition 8, a secure implementation can detect the modifications from the intruder. The test verdict of each transition trace is defined as follows.

**Definition 10 (test verdict).**

If *σ* ∈ *Tr*_*gen*_ is generated from protocol specification *S* and *M* is the protocol implementation, then one has the following:

- if *σ* contains any *modify* action of intruder, the test case *te* : {*σ* → *fail*∣*Out*(*σ*, *S*) = *Out*(*σ*, *M*)};
- if *σ* does not contain any *modify* actions, the test case *te* : {*σ* → *pass*∣*Out*(*σ*, *S*) = *Out*(*σ*, *M*)};
- otherwise, the test verdict is uncertain.

Then the security test cases to verify the protocol implementations are generated. For example, by using our algorithm, 304 test cases are generated to verify the security of NSPK protocol implementations. In Table 1, we present 5 security test cases as an example. As we know, the NSPK protocol has been proved insecure with the attacks of MITM; one possible attack strategy has been proposed by Gavin Lowe in 1995 and this attack strategy corresponds to Test 4 of our example.

## 5. Conclusion and Future Works

In this paper, we proposed an extension model of the classic IOLTS for the purpose of security verification of protocol implementation. This extended model defines the nonnegligible security properties with partial labels to identify the security actions. Then by proposing the intruder and the combination model, the security protocol can be modeled. The proposed model can help users to generate the probable transition sequences which contain the interactions between intruder and the protocol implementations. Those generated sequences are the abstract message scenarios to verify the implementations of security protocol. We also presented a corresponding test generation algorithm to automatically generate the test cases. Some test cases of the NSPK protocol are presented also and the MITM attack of NSPK is verified to be concluded in our test cases. In the future work, we plan to add the fuzzy sets during the test generation process, which can better simulate the actions of intruder of a network system. Some real network protocols, such as RADIUS and the protocol used in [26], are going to be considered. And the more complex experiments [27] are going to be defined.

## Figures and Tables

**Algorithm 1:**
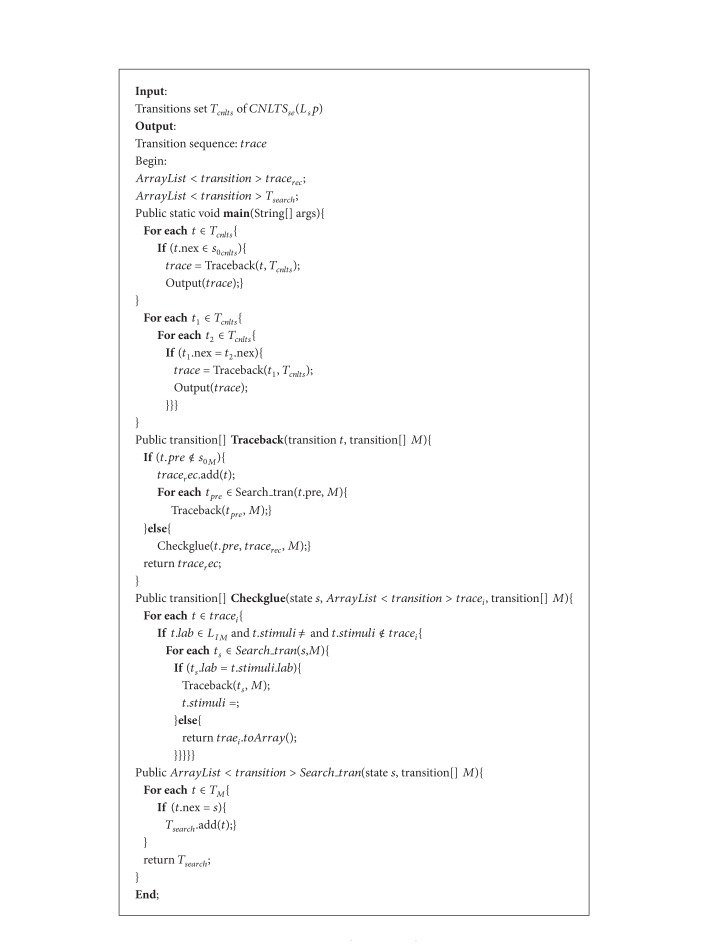
Test generation algorithm.

**Table 1:** Some security test cases for NSPK protocol.

Test 1	?Dest, !Ask, ?Ask, ?sid, modify, !sid', ?rid, forward, !rid, ?cipher, forward, !cip, !Ask', ?Ask, ?sender, *τ*_0__*r*_, ?receiver, *τ*_1__*r*_, ?cipher, *τ*_2__*r*_, !Rpl, ?Rpl, ?sid, forward, !sid, ?rid, forward, !rid, ?cipher, forward, !cip, !Rpl, ?Rpl, ¬sender, fail;

Test 2	?Dest, !Ask, ?Ask, ?sid, forward, !sid', ?rid, forward, !rid, ?cipher, forward, !cip, !Ask', ?Ask, ?sender, *τ*_0__*r*_, ?receiver, *τ*_1__*r*_, ?cipher, *τ*_2__*r*_, !Rpl, ?Rpl, ?sid, forward, !sid, ?rid, forward, !rid, ?cipher, forward, !cip, !Rpl, ?Rpl, ¬sender, pass;

Test 3	?Dest, !Ask, ?Ask, ?sid, forward, !sid, ?rid, forward, !rid, ?cipher, forward, !cip, !Ask, ?Ask, ?sender, *τ*_0__*r*_, ?receiver, *τ*_1__*r*_, ?cipher, *τ*_2__*r*_, !Rpl, ?Rpl, ?sid, forward, !sid, ?rid, forward, !rid, ?cipher, forward, !cip, !Rpl, ?Rpl, ?sender, *τ*_0__*i*_, ?receiver, *τ*_1__*i*_, ?cipher, *τ*_2__*i*_, *τ*_3__*i*_, !Cfm, ?sid, forward, !sid, ?rid, forward, !rid, ?cipher, forward, !cip, !Cfm, ?Cfm, ?sender, *τ*_3__*r*_, ?receive, *τ*_1__*r*_, ?cipher, *τ*_2__*r*_, *τ*_4__*r*_, pass;

Test 4	?Dest, !Ask, ?Ask, ?sid, modify, !sid', ?rid, forward, !rid, ?cipher, forward, !cip, !Ask', ?Ask, ?sender, *τ*_0__*r*_, ?receiver, *τ*_1__*r*_, ?cipher, *τ*_2__*r*_, !Rpl, ?Rpl, ?sid, modify, !sid', ?rid, modify, !rid', ?cipher, modify, !cip', !Rpl', ?Rpl, ?sender, *τ*_0__*i*_, ?receiver, *τ*_1__*i*_, ?cipher, *τ*_2__*i*_, *τ*_3__*i*_, !Cfm, ?sid, modify, !sid', ?rid, modify, !rid', ?cipher, modify, !cip', !Cfm', ?Cfm, ?sender, *τ*_3__*r*_, ?receive, *τ*_1__*r*_, ?cipher, *τ*_2__*r*_, *τ*_4__*r*_, fail;

Test 5	?Dest, !Ask, ?Ask, ?sid, forward, !sid, ?rid, forward, !rid, ?cipher, forward, !cip, !Ask, ?Ask, ?sender, *τ*_0__*r*_, ?receiver, *τ*_1__*r*_, ?cipher, *τ*_2__*r*_, !Rpl, ?Rpl, ?sid, forward, !sid, ?rid, forward, !rid, ?cipher, forward, !cip, !Rpl, ?Rpl, ?sender, *τ*_0__*i*_, ?receiver, *τ*_1__*i*_, ?cipher, *τ*_2__*i*_, *τ*_3__*i*_, !Cfm, ?sid, modify, !sid', ?rid, forward, !rid, ?cipher, forward, !cip, !Cfm', ?Cfm, ?sender, *τ*_3__*r*_, ?receive, *τ*_1__*r*_, ?cipher, *τ*_2__*r*_, *τ*_4__*r*_, fail;
